# Understanding drug uptake and binding within targeted disease micro-environments in patients: a new tool for translational medicine

**DOI:** 10.1186/2001-1326-1-8

**Published:** 2012-05-31

**Authors:** György Marko-Varga, Ákos Végvári, Melinda Rezeli, Kaiu Prikk, Peeter Ross, Magnus Dahlbäck, Goutham Edula, Ruth Sepper, Thomas E Fehniger

**Affiliations:** 1 Clinical Protein Science & Imaging, Biomedical Center, Dept. of Measurement Technology and Industrial Electrical Engineering, Lund University, BMC C13, SE-221 84, Lund, Sweden; 2 First Department of Surgery, Tokyo Medical University, 6-7-1 Nishishinjiku Shinjiku-ku, Tokyo, 160-0023, Japan; 3 Institute of Clinical Medicine, Tallinn University of Technology, Akadeemia tee 15, 12618, Tallinn, Estonia; 4 AstraZeneca R&D Lund, Lund, Sweden

**Keywords:** Clinical drug administration, Ipratropium bromide, Bronchial tissue, MALDI-MS Imaging, MALDI LTQ Orbitrap XL

## Abstract

**Background:**

For many common global diseases, such as cancer, diabetes, neurodegenerative and cardiovascular diseases there is an unmet need for diagnosing early indications of disease that could enable medical intervention and early treatment. The treatment of these diseases will require detailed knowledge of targeted pathways involved in disease pathogenesis but also the mode of drug actions at the biological location on these targets. Translational medicine is a new area of research where expert from different disciplines involved in basic science and clinical disciplines meet and join forces. Mode-of-drug-action mechanisms elucidation is key in the characterization of drugs that can relate to both efficacy and safety.

**Methods:**

Matrix assisted laser desorption/ionization mass spectrometry imaging (MALDI-MSI) was used providing evidence into the fate (destinations and distributions) of administered drugs within tumor regions of lung compartments.

**Results:**

We hereby present a pulmonary study in which we have isolated lung tissue after inhaled drug administration and then localized the drug within airway wall compartments. The histology also provides evidence of drug binding to smooth muscle cell microenvironments. We also identified lung tissue regions with tumor cell invasion in these COPD patients.

**Conclusions:**

The ultimate goal is to identify bridging comprehension that forms a knowledge base that can be used by society to develop a better treatment and medicine for patients. Our results demonstrated that robust imaging data could be generated confirming drug localization in pulmonary regions of COPD patients with tumor pathology.

**Trial registration:**

Tallinn Medical Research Ethical Committee decision #1724, 18.06.2009

## Background

Translational medicine (TM) is not really a new research area, but rather a compilation of a number of research areas that the scientific community has been involved in for the last decades, in the clinic, academia as well as industry. TM is in general referred to as the medical practice that builds on a work-flow that involves evidence-based medicine derived from linking knowledge about disease pathways observed clinically with laboratory models of disease that eventually develops into new intervention and treatment paradigms [1].

Personalized medicine is in this context linked to a targeted treatment: where a protein target is selected that has key regulating roles within a specific disease [2-5].

TM is a research area that integrates basic research as well as social and societal infrastructures where the goal is to improve and deliver optimal patient care [6,7]. In this respect, the concept of 4P Medicine has been introduced: preventive measures which may extend beyond primary healthcare services to preventive measures limiting disease onset [8]. The translational area is highly timely, as the modern healthcare area worldwide has many shortcomings in terms of efficiency, both for the patient as well as for the community that needs to bear the total cost for care.

By directing TM into the pulmonary diseases, we have been actively driving these developments for the last decade, where the starting point for us has been the pathophysiology and disease [9-17].

This is especially relevant to diseases such as lung cancer and chronic obstructive pulmonary disease (COPD), the latter, a disease that is rapidly increasing, and that present itself in combination with lung cancer. These pulmonary diseases currently carry a huge mortality and significant cost to the healthcare system. Cigarette smoking is the most important risk factor in the development of a number of major life threatening diseases including cancer, COPD, coronary heart disease and stroke. As an example, smoking prevalence in Estonia, EU in adults in 2005 was 31.5 % (42 % in male, 21 % in female) and in youth 24 % (boys 30 % and girls 18 % from age [15-18]. These numbers have continued to increase since that time. Quite often patients develop clinical complaints related to smoking long after pathological damage has begun. Disease related to smoking is heterogeneous within individual patients with wide variety of co-morbidity involving any and all of the above-mentioned disorders. The time frame required for disease development also differs between individuals by smoking history, age, gender, diet, genetic background, and environmental exposure. The staging of disease is most often determined by clinical symptoms using a variety of measurement devices.

The big challenge dealing with translational science is to be able to align and correlate the predictions of disease biology and possible treatment windows seen in animal models with disease presentations and treatments available in everyday clinical practice. The phenotyping of individual disease presentations within the range of spectrums of possible diseases will be a key component of aligning effective therapy and in developing novel drugs. It is in this arena that TM will have its future opportunities to bring ideas, innovations and best clinical practice into an organized research structure. A structure that utilizes the advanced platforms of science and technology that is coupled to a broad base of expertise that crosses over between disciplines. The novelty will be to perform the work along the lines of a new direction in thinking, where the boundaries allow for a focused translation of our understanding of the mechanisms that change healthy biology into diseased, and the ways of impacting on these changes with targeted drug treatment intervention [18].The current study provides new insights to previously published work, with a drug localization that is targeted towards smooth muscle cell regions. In addition, this study also delivers imaging data that provides evidence of tumor regions of lung compartments and MALDI-MSI selectivity and precision.

## Methods

### Patients and clinical material

The bronchial tissue samples were biopsied from patients appointed to fiber optic bronchoscopy for different clinical indications. In total, five patients were included in this study, and phenotypes and diagnosis were as follows: two patients with suspicion to bronchial carcinoma, one for chronic obstructive bronchitis with exacerbation, and two with sub-febrile body temperature with unknown reason. Inform consent was obtained from all patients (Tallinn Medical Research Ethical Committee decision #1724, from 18.06.2009). Before routine bronchoscopy 1 mL of Berodual (Boehringer Ingelheim Int. GmbH, Germany) solution, containing 250 μg/mL ipratropium bromide (systematic (IUPAC) name, [8-methyl-8-(1-methylethyl)-8-azoniabicyclo[3.2.1] oct-3-yl] 3-hydroxy-2-phenyl-propanoate) together with fenoterol hydrochloride 500 μg/mL was administrated in the nebulized form via PARI TurboBOY® inhalation device (total output rate 500 mg/min, with MMD 3.5 μm and 68 % mass percentage below 5 μm, PARI GmbH) with nebulized medium of 0.9 % NaCl (5 mL) and inspiratory flow 20 L/min for 10 minutes (http://www.pari.de). After endobronchial inspection with the white-light (WL) and autofluorescence image (AFI) bronchoscope (Pentax) forceps (Olympos) bronchial biopsies were obtained from right upper lobe *carina* followed by slow freezing over two minutes by floating in a bath of super-cooled with dry ice isopentane. Bronchial tissue samples were then stored at −70 °C until sectioning.

### Tissue sectioning and matrix application

Cryostat thin slices of 10-μm thickness were prepared using a Leica CM5030 cryostat. Serial sections were placed upon frosted end microscope slides (Menzel-Gläser) and air-dried. Then 500 μL of 7.5 mg/mL α-cyano-4-hydroxycinnamic acid (CHCA) matrix solution in 50 % acetonitrile/0.1 % trifluoroacetic acid was sprayed stepwise onto the tissue sections by using an Aztek airbrush model A4709 (Testor Corp., Rockford, IL), releasing short puffs of matrix solution interrupted with 3–5 s dwell time. Tissue sections prepared with matrix were allowed to dry at room temperature in vacuum for an hour prior to MALDI-MS analysis.

### MALDI-MS drug characterization and imaging analysis

A MALDI LTQ Orbitrap XL mass spectrometer (Thermo Scientific, Bremen, Germany), equipped with a 60 Hz 337 nm nitrogen pulse laser (LTB Lasertechnik Berlin GmbH, Berlin, Germany), was used in this study for compound tissue imaging. Imaging of drugs was performed in both MS and MS/MS data collection in positive mode, sampling tissue sections with 30 μm raster arrays without laser movement in measuring position. The FT mass analyzer (Orbitrap) was utilized at 60,000 resolution collecting spectral data in the mass range of 250–1000 Da generated by 20 laser shots at 10 μJ. The linear ion trap analyzer was operated in the 90–500 Da mass range and utilized to fragment the parent ion of ipratropium (*m/z* 332.332) at normal scan rate, isolating the ions in *m/z* 3.0 width. Normalized collision energy was 50 % during an activation time of 30 ms and activation *Q* of 0.250 was applied with wideband activation.

The ImageQuest™ software (Thermo Scientific, San José, CA) was used to create reference optical images of the sample including the region of interest outlined for analysis of the drug parent and fragment ions.

**Figure 1:**
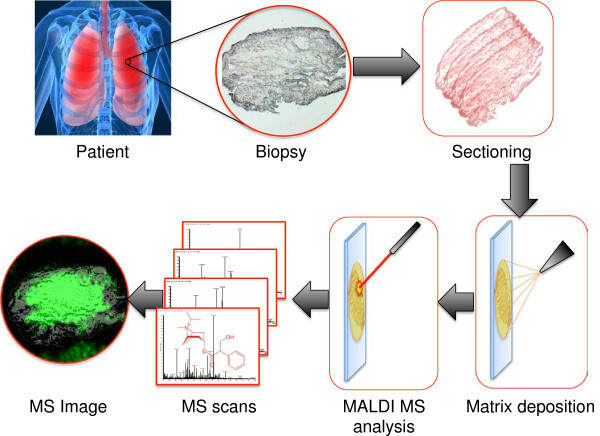
Illustration presenting the experimental workflow of MALDI-MS imaging analyzing drug levels in patient lung tissues.

The overall experimental strategy that was developed within the study is illustrated in Figure 1.

## Results and discussions

Linking disease phenotypes with diagnosis and translational predictions.

It is clear from a historical background that in the future biomedical sciences will be driven by the ability to adopt novel technologies, which will in turn generate huge amounts of data outputs from clinical samples. One major consequence will be to utilize the new technology deliveries as the basis to understand the disease complexity and to develop new treatments. COPD is a major concern in the world because of the high value of smoking prevalence rate. Clinically these patients experience breathlessness, productive cough, shortage in many life functions especially those associated with constitutional capability. The diagnostic tools currently address structural (CT lung density scan, CT airway wall thickness) and functional abnormalities (spirometry for expiratory flow, residual volume (RV), total lung capacity (TLC), forced residual capacity (FRV), and airway specific resistance and conductance). These diagnostic approaches are valid in the more advanced COPD leaving, however, out the early onset of lung functions decline in chronic smokers who destined to but have not yet developed irreversible changes in lung structure and function. The development of new protein biomarker assays that could diagnose early ongoing disease greatly assist here and add context to and complement the existing diagnostic tools. The CT scans that are typically used in lung density measurements in smokers will provide information on the distribution of emphysema, but will not differentiate between older inactive emphysema lesions and active areas of current parenchyma destruction. Protein measurements capable of providing quantitative information on current ongoing lung tissue matrix destruction would be invaluable assets in monitoring progression of disease in the context of the CT scans measuring emphysema and the functional tests.

Over recent time, it has been demonstrated that early detection, prescription of personalized medicine based on disease type, and evaluation of response to treatment has significantly impacted on advancing outcomes, and reducing cost to healthcare systems. These diseases are known to be highly complex and multifactorial. It is not possible at this stage, to assign a single molecule related to one disease or clinical complaint. On the contrary there are hundreds (multiple signals), that relate to a change of a normal state to a diseased. Consequently, there is a need of selecting the key regulators from multiple signals. This is a highly demanding task, as this is hampered by the lack of tools and data for defining the biological mechanisms involved in the early onset of establishing of disease development. This is probably the biggest challenge within the field of translational medicine.

In addition, the modeling of disease progression and the evaluation of treatment response are also active areas within the science community. Lung cancer and COPD are both known to cluster in families and are more common in elderly population. Aggregation has been observed in families which would suggest a genetic or an environmental connection.

**Figure 2:**
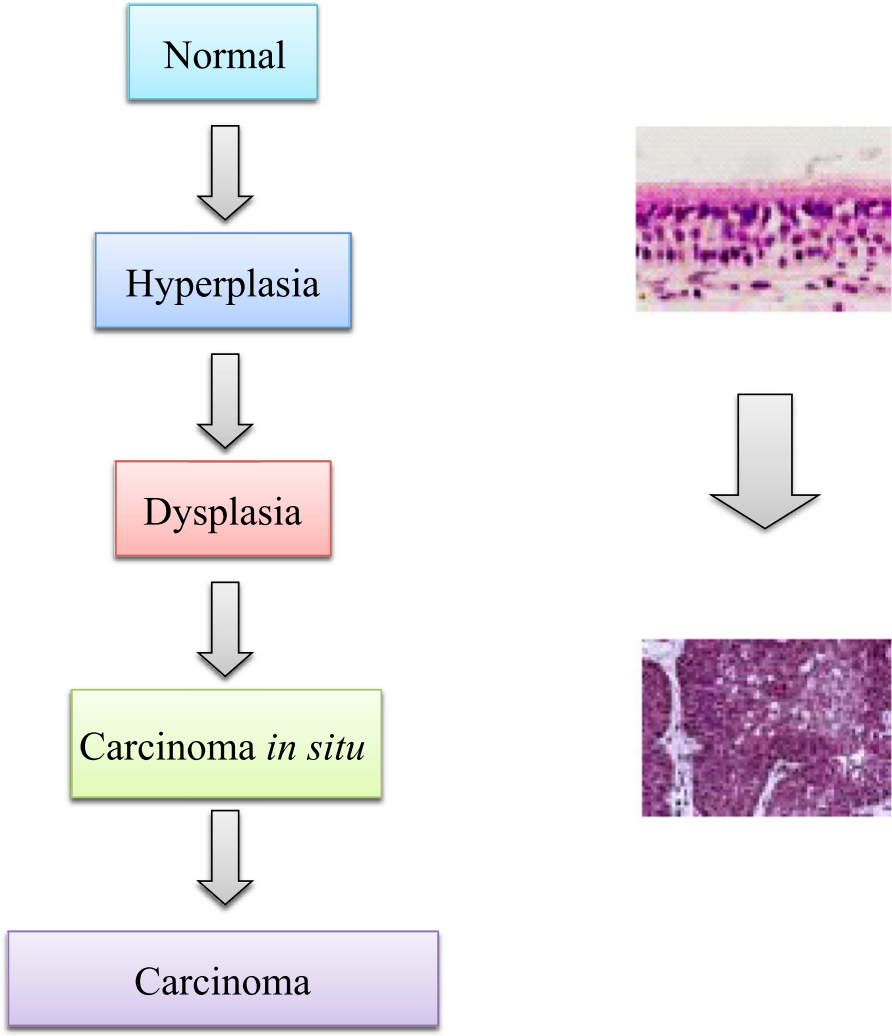
Schematic illustration of pulmonary disease progressions.

Clinically and pathologically it has been observed that smokers with COPD often develop lung cancer. The transition between normal epithelial cell biology and transformation into a malignant cell status can be defined histologically (Figure 2), as morphological changes developing hyperplasia, followed by dysplasia and a final diagnosis of carcinoma. Diagnostically, these changes can be observed microscopically at pathology examination but also macroscopically in situ at the sites of disease by changes in fluorescent patterns seen by endoscopy [19,20].

Currently these disease areas are facing significant challenges where major research resources are directed, such as:

1. Stratifying large and increasing number of lung cancer phenotypes and their link to COPD

2. Diagnosis, at an early stage, where limited markers are available, with a need for potential targeted medical opportunities

3. Lack of monitoring of any given treatment’s effectiveness

4. Correlation between in-situ molecular mechanism of the disease and peripheral molecular changes

5. Methods to measure at a molecular level what changes have occurred at sites of targeted drug intervention.

The optimization of treatment, based on individual medical need is currently a fundamental cornerstone to the rebuilding of the entire medical and clinical system. In this respect, the concept of personalized medicine has been established as a standard working proposition worldwide. On the technology side, a major and unremitting effort is underway to achieve these developments such as the requirement to establish the standard ranges of expression in quantitative assays. These assays need to be able to separate healthy from diseased individuals in a variety of basic sciences such as genomics, proteomics, and metabolomics, as well as clinical sciences. Clinically, surveillance, diagnosis and treatment are in focus, which are in turn applied to match scenarios of individual disease with best practice treatment efficacy at the level of each individual patient. Lately, a major focus of the introduction of targeted personalized medicine is marker associations with drug efficacy and safety. In this respect, the interface that relates the localization of drugs and metabolites that can be related to pharmacokinetic-, and pharmaco-dynamic data will form the basis for optimal conditions whereby drug dosing and delivery could be made. In particular, targeted drug treatments, that is directed towards a specific patient groups will benefit from a predictive guidance utilizing matrix assisted laser desorption/ionization mass spectrometry imaging (MALDI-MSI). As mass spectrometry imaging does not require any chemical labeling, the “cold compound” has the great advantage to provide data that can be directly linked to the pharmacological effects of the drug.

### Drug localization by MALDI-MS imaging

For many drug therapies, there is little knowledge about the ultimate distribution patterns of the compounds within tissue compartments following treatment. It is possible to label drugs with a tracer and follow their uptake using technologies such as positron-emission tomography (PET) and autoradiography. For both of these methods the physical manipulation of the compound by the labeling could change the properties of the compound. Over the last decade a method to identify unlabeled drugs in tissue has been under development using MALDI-MSI. With MALDI-MSI continual incremental sampling can be performed upon tissue taken directly from the body to identify cellular locations that contain the specific drug ion signatures of the compound in question [21].

**Figure 3:**
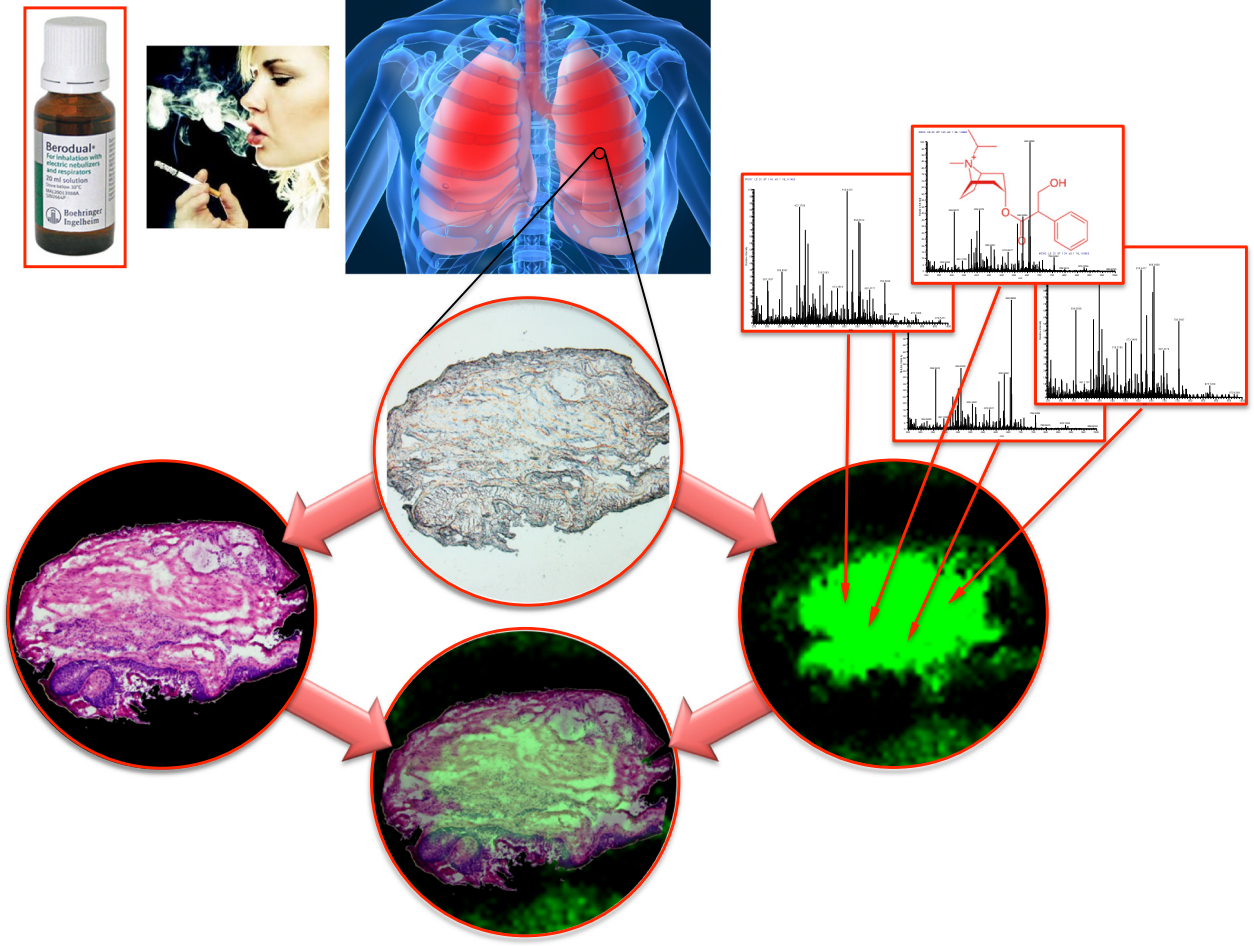
Illustration of translation medicine and its impact on novel drug development and protein biomarker diagnostics.

**Figure 4:**
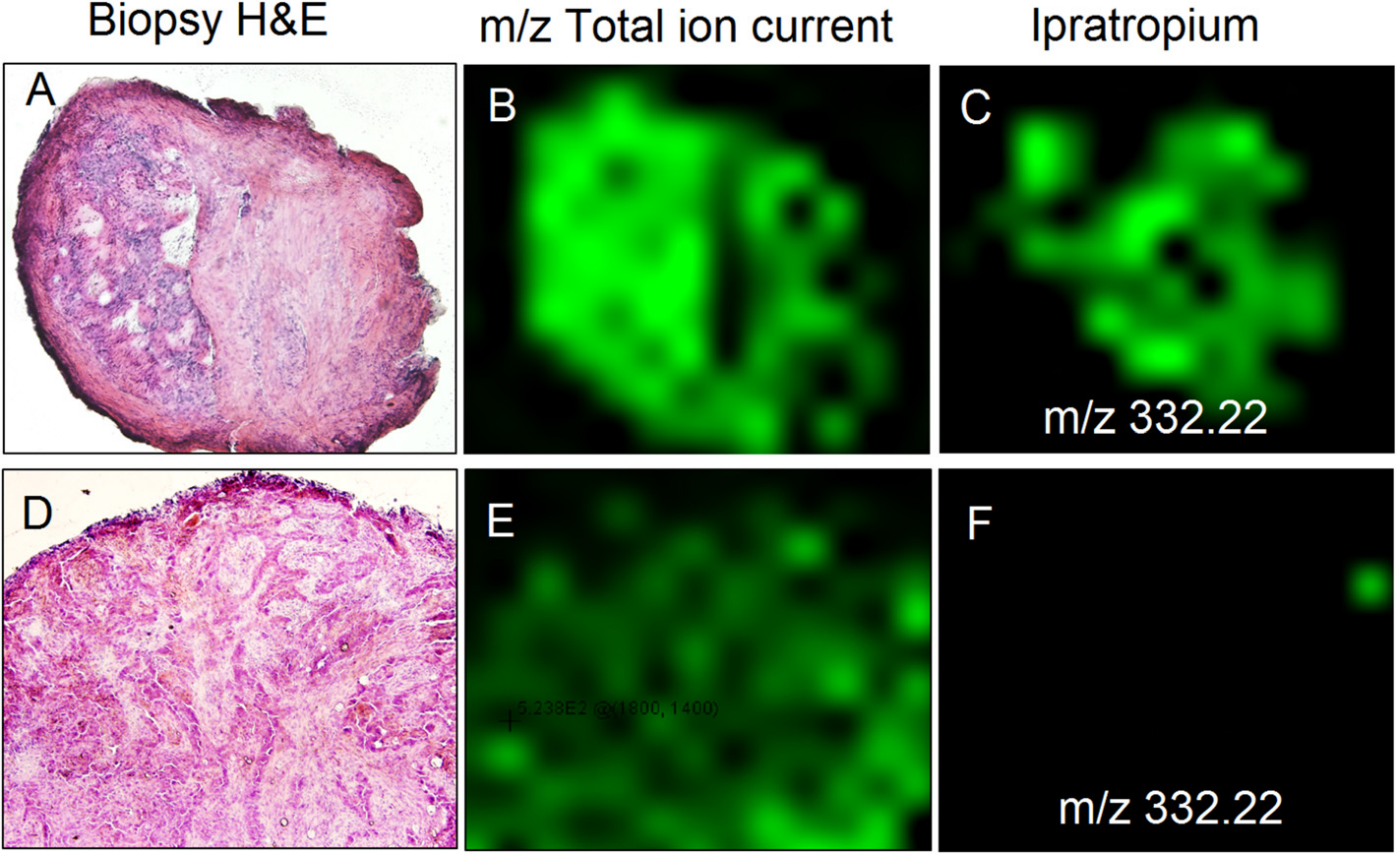
**Airway wall biopsies of two subjects treated with inhaled ipratropium detected by MALI-MSI.** One sample (**A**-**C**) with intact airway wall showed specific ipratropium mass fingerprint (*m/z* 332.22) while subject with airway tumor (**D**-**F**) showed no drug uptake.

We recently performed a proof-of-principle in order to test the power of applying MALDI-MSI to demonstrate the qualitative drug distribution within pulmonary microenvironments. The concept and mass spectrometry imaging is illustrated in Figure 3. This is the first reported study in man, at a resolving power of 30 μm, of drug localization within organ compartments using normal clinical dosing schemes and standardized laboratory measurement. We mapped the occurrence of an inhaled bronchodilator, the muscarinic receptor antagonist ipratropium, within human bronchial biopsies obtained by fiber optic bronchoscopy shortly after dosing exposure [22]. Samples coated with a matrix compound were analyzed by a MALDI LTQ Orbitrap XL mass spectrometer at a resolving power of 30 μm, spatial resolution. Ipratropium parent ion (*m/z* 332.332) and daughter ions (*m/z* 166.2 and 290.2) were coincidently partitioned within the sub mucosal spaces containing targeted airway smooth muscle in 4/5 subjects. We could conclude from our investigations that ipratropium is rapidly absorbed into the airway wall. Of interest here was that the airway biopsy not showing ipratropium uptake was histologically found to represent a tumor forming within the airway wall (see Figure 4). The limited drug uptake in the tumor could be due to a variety of reasons including defects in drug transport and changes in muscarinic receptor density within the effected tissue microenvironment. The ability to discriminate between positive and negative examples of drug uptake provides a very powerful tool in understanding drug efficacy.

We could also conclude from our study that the specific region of tissue isolation (from the patient presented in Figure 4, where the tumor growth was identified), that the tumor could be visualized in real-time upon endobronchial biopsy isolation.

**Figure 5:**
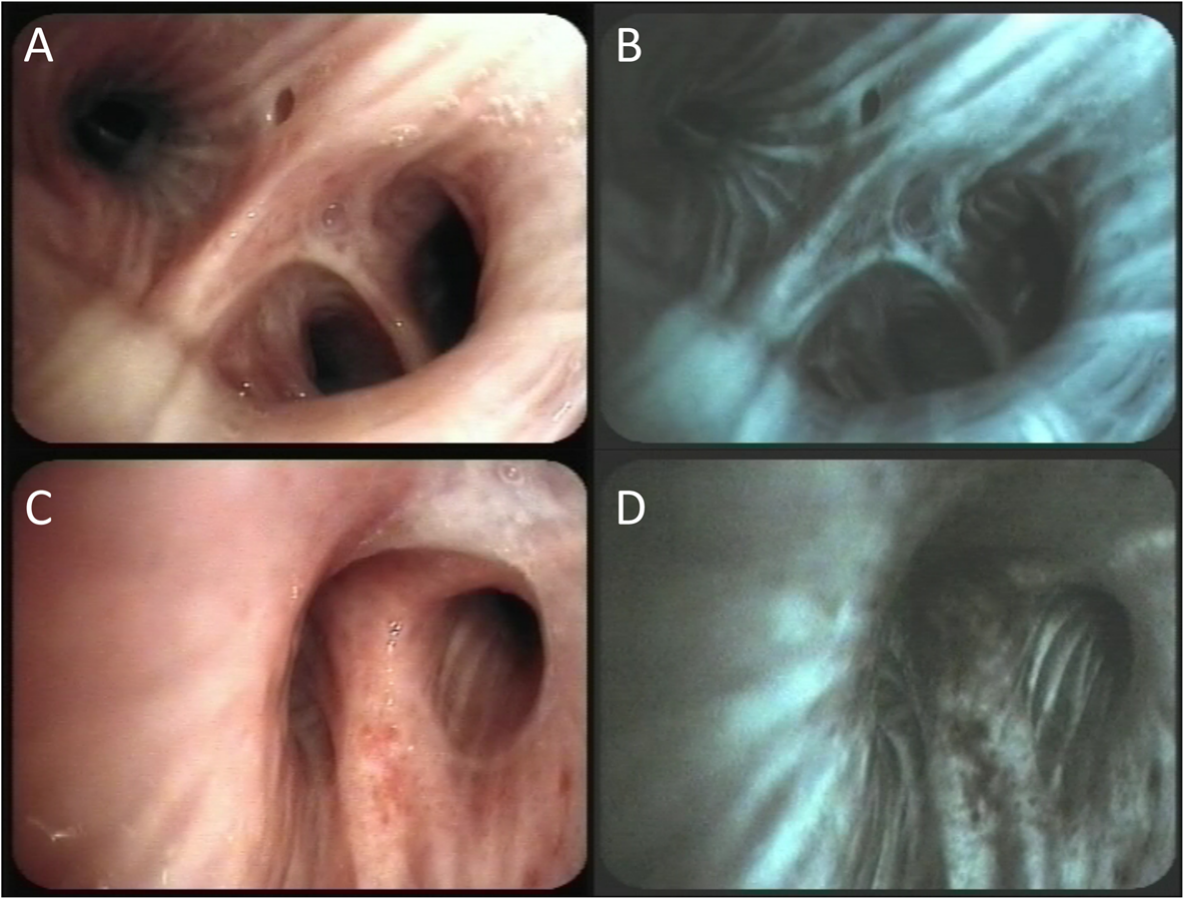
**Images showing the endobronchial images of chronic obstructive endobronchitis with white light (A) and autofluorescence (B) bronchoscopy.** Endobronchial images of bronchial carcinoma at the right upper lobe shown with white light (**C**) and autofluorescence (**D**) bronchoscopy.

With endobronchitis imaging, using the white light source in comparison to autofluorescence, tumor differentiation can be made, by identifying the tumor regions within microenvironments of the bronchi. Figure 5 provides a nice example and illustration of a healthy endobronchial image using white light (Figure 5A) in relation to the autofluorescence bronchoscopy (Figure 5B). The COPD patient, with tumor growth, is illustrated in Figure 5C in relation to Figure 5D, that provides evidence of the tumor region. This can be seen as a white region on the upper regions of image capture Figure 5C, in comparison to the autofluorescence image (Figure 5D), where this white region comes up as a black area. This is the ultimate differential read-out that is used for tumor diagnosis on a routine basis by clinicians.

**Figure 6:**
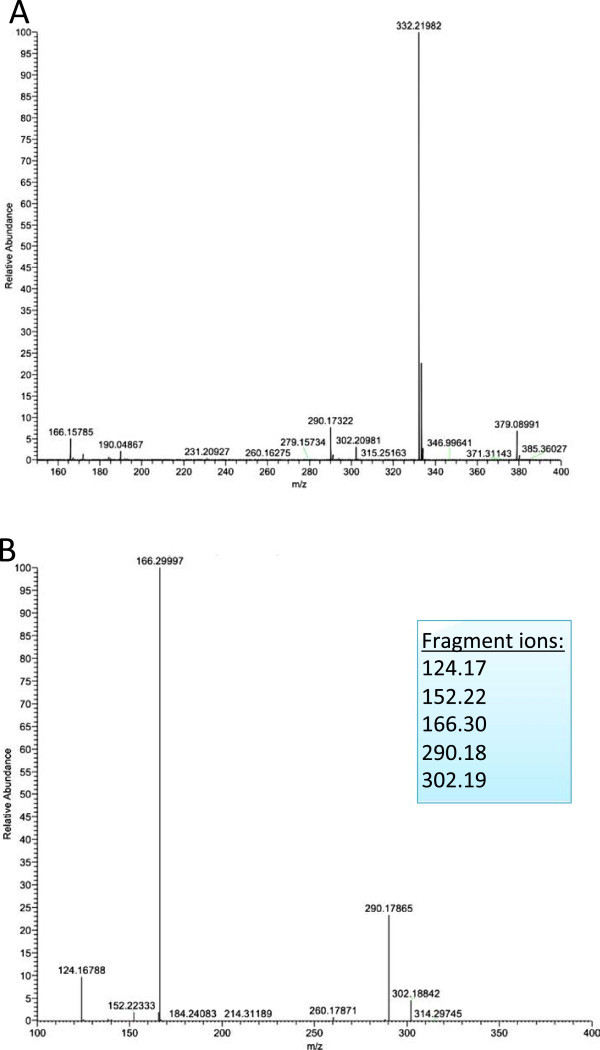
**Ipratropium identification within the pulmonary compartment with smooth muscle cells: (A) parent ion (*m/z*332.332) and (B) fragmentation pattern (*m/z*124, 17, 152, 22 166.2 and 290.2).**

The theoretical target of ipratropium was the muscarinic receptors M1-M3 that are expressed differentially on smooth muscle cells and inflammatory cells. Our results showed that the ipratropium signal (typical mass spectra of ipratropium in lung tissue presented in Figure 6) could be observed in areas of the biopsy that coincidently contained either or both, smooth muscle cells and/or inflammatory cells. Our current work is characterizing the expression of the specific muscarinic receptor in this material using immunohistochemistry. This is a powerful advantage of the MALDI-MSI technique in that the same tissue section that has been used to quantify drugs can be used in additional assays to characterize the microenvironment.

## Conclusions

Looking into the pipeline of drug products in development and currently in clinical phase trials, the expectations of new treatments using Personalized Medicine approaches of targeted medicines using either small molecules as well as antibody-based biopharmaceuticals are expected to grow considerably. It is envisioned that the upcoming generation of personalized drugs for targeted and stratified patient treatment will break through in major disease areas such as, lifestyle-related cancers, in particular lung cancers that has the highest mortality including a the co-incident disorder chronic obstructive pulmonary disease.

Mass spectrometric technologies can provide the “phenotypic fingerprint” required for the concept of Personalized Medicine. Mass spectrometry-driven target biomarker diagnoses in combination with high resolution computed tomography could provide a critical pathway initiative facilitated by a fully integrated e-Health infrastructure system. By building experimental drug binding property characteristics, prediction platforms can be established that defines the “possible treatment windows”.

The recent developments and announcement from the Human Proteome Organization (HUPO), on the Human Proteome Project (HPP) is a major undertaking [23].

## Abbreviations

TM, Translational medicine; COPD, Chronic obstructive pulmonary disease; CT, Computer tomography; RV, Residual volume; TLC, Total lung capacity; FRV, Forced residual capacity; PET, Positron-emission tomography; MALDI-MSI, Matrix assisted laser desorption/ionization mass spectrometry imaging; HUPO, Human proteome organization; HPP, Human proteome project.

## Competing interest

The authors declare that they have no commercial or non-financial competing interest.

## Authors’ contributions

GMV, MD GE, TF, and PR designed the clinical study and outline, wrote the majority of the manuscript, AV and MR and TF performed the experimental work of the clinical materials and calculations, KP and RS performed the clinical parts, including drug delivery, imaging and biopsy isolations. All authors read and approved the final manuscript.
